# Coronary Heart Disease and ABO Blood Group in Diabetic Women: A Case-Control Study

**DOI:** 10.1038/s41598-019-43890-4

**Published:** 2019-05-15

**Authors:** Seyyed Hasan langari, Adele Bahar, Leila Asadian, Saeid Abediankenai, Seyed Shojaeddin Namazi, Zahra Kashi

**Affiliations:** 1 Student Research Committee, Mazandaran University of Medical Sciences, Sari, Iran; 2 Radiology department, faculty of medicine, Shahid Beheshti University of Medical Sciences, Tehran, Iran; 3 Diabetes Research Center, Mazandaran University of Medical Sciences, Sari, Iran; 4 Emergency Medicine department, faculty of medicine,Student Research committee, Mazandaran University of Medical Sciences, Sari, Iran; 5 Immunogenetics Research Center, Mazandaran University of Medical Sciences, Sari, Iran; 6 Cardiovascular Research Center, Faculty of Medicine, Mazandaran University of Medical Sciences, Sari, Iran

**Keywords:** Physiology, Cardiology, Type 2 diabetes

## Abstract

Numerous investigations conducted in general population have reported that certain ABO blood group may increase the risk of coronary heart disease (CHD). However, this association has not been yet well established and even is less clear in diabetic patients. Considering that women with type 2 diabetes mellitus (T2DM) are at greater risk to develop CHD and have higher cardiovascular mortality, this study aimed to evaluate the association between CHD and ABO blood group in women with T2DM. A case control study of eight hundred eighty-one (881) diabetic women was enrolled in this study. Among them, two hundred thirty eight (238) patients were identified to have CHD (CHD+) and two hundred eighty two (282) of them were identified without CHD but matched with the first group for other CHD risk factors (CHD−). ABO blood type (A, B, AB, O, and Rhesus factor) for both groups were determined. To compare the magnitude of the correlation between various blood groups with CHD development, odd ratios (OR) with 95% confidence intervals (CI) was calculated. Our results demonstrates that the percentage of AB blood group was significantly higher in the diabetic women with concurrent CHD than in those without CHD [30 (12.7%) *vs*. 13 (4.6%), Odd ratio: 2.9 (95%CI: 1.5–5.7), P = 0.001]. The results of the present study clearly demonstrate that the AB blood group has a higher odd ratio for the development of CHD and can be considered as a risk factor for the development of CHD in females with T2DM. More comprehensive studies are required to confirm these results.

## Introduction

Coronary heart disease (CHD) is more common among patients with type 2 diabetes mellitus (T2DM)^1,2^. The male sex is considered an important risk factor for CHD in the general population, but studies on diabetic patients suggest that diabetic women have a higher risk of developing CHD^3,4^. In addition, women with concomitant T2DM and CHD achieve LDL-C goals less than men do^5^. In fact, cardiovascular mortality is almost doubled in diabetic women compared to men^6^. There are several risk factors attributed to CHD such as hypertension, family history of CHD, obesity, cigarette smoking, older age, and lipid abnormalities^2,7^. Some of the risk factors are shared between diabetes mellitus and CHD, which can potentiate comorbidity of these two non-communicable disorders. In addition, diabetes mellitus, itself, is a proven risk factor for CHD^8^. Given that CHD is the major cause of mortality in diabetic patients^9^, seeking for other probable risk factors to develop CHD in diabetic patients is of utmost importance.

Numerous investigations have reported that certain ABO blood group can increase people’s susceptibility to some disorders, including cancers and infectious diseases^6–8^. Various investigations have also been conducted to determine the role of ABO blood group on CHD development. In some studies, conducted in the general population, non-O blood groups were identified as potential risk factor for the development of cardiovascular diseases. Moreover, CHD was less common among individuals with the O blood group^9–11^. In addition, some studies have reported a significant association between the A and B alleles and cardiovascular disease^11,12^. Although higher prevalence rates of T2DM have been reported among individuals with certain blood groups^13^, we could not find any research that specifically evaluates the role of ABO blood groups in the development of CHD in diabetic patients.

Due to the close comorbidity of T2DM and CHD, especially in female patients, this study was designed to evaluate, for the first time, the association between ABO blood groups and the development of CHD among diabetic women in Iran.

## Methods

This case control study was conducted among women with T2DM living in the north of Iran in the Mazandaran province, city of Sari. Eight hundred and eighty-one (881) outpatient diabetic women referred to the academic diabetes clinic of ‘Mazandaran University of Medical Sciences’, between March 2015 to January 2016. They were selected using the purposive sampling method. To diagnose T2DM, the American Diabetes Association criteria were employed. Additionally, individuals who were taking medications for their T2DM at the time were also enrolled in the study. The student research committee and ethics committee of ‘Mazandaran University of medical sciences’ approved the research protocol (code 94–277). The informed consent was obtained from all participants.

All enrolled subjects were evaluated for CHD. Diagnostic methods were based on the American College of Cardiology Foundation and the American Heart Association (ACCF/AHA) guidelines and regulations published and updated for the diagnosis and management of patients with stable ischemic heart disease (SIHD) in 2012 and 2014^14,15^. Some patients had documented CHD: previous myocardial infarction, coronary artery bypass graft (CABG), coronary stent placement, balloon angioplasty, or just angiographically confirmed coronary artery disease. The patients without documented CHD were referred to a cardiologist for CHD evaluation. At first a functional study (exercise stress test or radionuclide myocardial perfusion imaging for patients who were unable to exercise) was conducted. If the functional study results were positive, coronary angiography was performed.

The case group included diabetic women with CHD (CHD+) and the control group (CHD−) included diabetic women who had negative exercise or nuclear stress tests and/or normal angiography results, but had matching CHD risk factors (age, duration of diabetes, lipid profile, blood pressure, body mass index and smoking), with the case group. Pregnant patients, and those who had liver disease of different etiologies, malignancies, or serum creatinine levels higher than 2 mg/dl, were excluded from this study.

The standard, slide agglutination technique was performed to determine the ABO blood group in both case and control subjects.

### Statistical analysis

Normally distributed variables were presented as mean ± standard deviation (SD). Independent t-test and chi-square tests were used to compare the quantitative and qualitative variables between the two groups, respectively. Odds ratios (OR) with 95% confidence intervals (CI) were used to compare the magnitudes of the correlations of the various blood groups with CHD development. *P*-values < 0.05 were deemed statistically significant.

## Results

### Characteristics of the study population

Of the 881 diabetic women enrolled in this study, 238 were confirmed to have CHD and were considered as the case group (CHD+). CHD evaluation was negative in other 643 subjects. Among those, 282 subjects were matched with the CHD+ group for other cardiac risk factors such as: age, body mass index (BMI), systolic and diastolic blood pressure (SBP, DBP), hemoglobin, lipid profile, thyroid stimulating hormone (TSH), duration of T2DM, glycosylated hemoglobin (HbA1c) and plasma creatinine levels. These patients were considered as the control group (CHD−) (Table 1). The history of CHD in the first-degree relatives of the patients was not significantly different between the two groups (p = 0.871). The mean age and duration of diabetes in CHD+ and CHD− groups were 59.03 ± 9.19 vs 59.02 ± 9.35 years (p = 0.995) and 11.41 ± 7.27 vs 11.91 ± 5.92 years (p = 0.385) respectively. Fourteen patients (6.3%) in CHD+ group and sixteen patients (6.2%) in CHD− group were smokers (p = 0.961). None of the patients reported alcohol consumption. Thirty percent (30%) of patients in CHD+ group and 14.9% in CHD− group received insulin to control their blood glucose level (p = 0.729).

**Table 1:** Demographic data and baseline variables of the case and control groups.

Variable	CHD - (Mean ± SD)	CHD+ (Mean ± SD)	P-Value
BMI (Kg/m2)	29.90 ± 4.59	29.85 ± 4.29	0.221
SBP (mmHg)	135.27 ± 17.40	133.25 ± 14.83	0.154
DBP (mmHg)	76.65 ± 9.54	77.41 ± 7.74	0.317
Triglyceride (mg/dl)	226.02 ± 93.67	213.15 ± 118.85	0.178
High Density Lipoprotein (mg/dl)	45.20 ± 11.38	46.27 ± 10.34	0.263
Low Density Lipoprotein (mg/dl)	104.49 ± 33.64	109.26 ± 30.37	0.09
Cholesterol (mg/dl)	193.58 ± 43.18	196.71 ± 39.88	0.391
Hemoglobin (mg/dl)	12.26 ± 1.33	12.32 ± 1.27	0.617
FBS (mg/dl)	142.01 ± 55.42	137.04 ± 52.06	0.294
HbA1C (%)	7.94 ± 1.51	7.87 ± 1.55	0.594
TSH (IU/L)	2.56 ± 1.71	2.76 ± 1.89	0.228
Plasma Creatinine (mg/dl)	0.90 ± 0.18	0.88 ± 0.17	0.204

T2DM: Type two diabetes mellitus; DBP: diastolic blood pressure; SBP: systolic blood pressure; HbA1c: glycosylated hemoglobin; TSH: thyroid stimulating hormone; BMI: body mass index.

### ABO blood types in the CHD+ and CHD− groups

**Figure 1:**
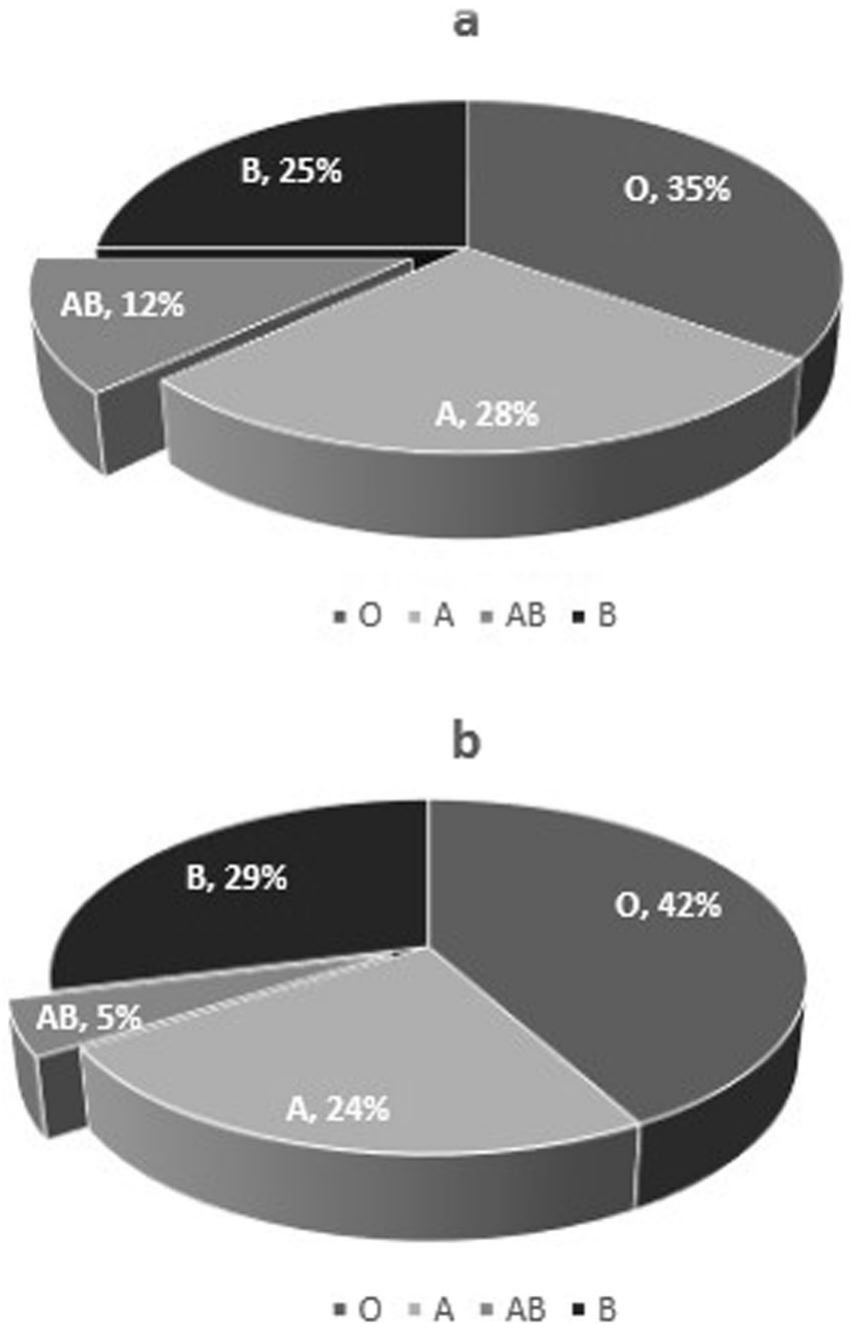
Blood group distribution in diabetic women with (**a**) and without (**b**) coronary heart disease.

Blood groups were determined using specific antibodies by agglutination method. The prevalence of blood group in the CHD+ and CHD− are shown in Fig. 1.

Our results showed that the percentage of AB blood group was statistically higher in the diabetic women with concurrent CHD than in those without CHD (12.3 vs. 4.7%). The odds of CHD occurring in diabetic women with AB blood group, compared to the odds (OR) of the CHD occurring in diabetic women with non-AB blood groups was 2.9 (CI: 1.5–5.7) (P = 0.001). The results showed no significant differences in the percentage of the other blood groups between CHD+ and CHD−groups. The percentage of the non-O blood groups was higher in the patients with CHD, however this difference did not reach significance (65 vs. 58.2%, P = 0.104). The percentage of the A and B blood groups in the CHD+ and CHD− groups were (27.8 vs. 24.1%, P = 0.304) and (24.9 vs. 29.4%, P = 0.236) respectively. The Rhesus factor (RH) was positive in 219 (92.0%) of the cases and 263 (93.3%) of the controls (P = 0.705).

### Association between AB blood group type and CHD risk factors

In order to determine the cause-association between the blood group and CHD, we compared other cardiac risk factors in CHD+ group between AB blood group and non-AB blood group (Table 2). We found that the systolic blood pressure was slightly higher in the AB blood group but this difference was not statistically significant (P = 0.065).There were no statistical differences in any other risk factors between the two groups.

**Table 2:** Baseline variables between the AB and non-AB blood groups in T”DM women.

Variable	Blood Group	Mean	SD	P Value
Age (year)	AB	58.19	7.93	0.536
	non-AB	59.12	9.39	
Duration of DM (year)	AB	12.37	7.53	0.486
	non-AB	11.62	6.49	
BMI (kg/m^2^)	AB non-AB	30.06 29.86	4.84 4.40	0.816
SBP (mm Hg)	AB non-AB	137.15 133.91	17.84 15.90	0.065
DBP (mm Hg)	AB non-AB	79.38 76.85	11.16 8.33	0.78
Triglyceride (mg/dl)	AB	224.40	106.50	0.734
	non-AB	218.53	108.44	
Cholesterol (mg/dl)	AB	190.23	37.23	0.404
	non-AB	195.74	41.77	
High Density Lipoprotein (mg/dl)	AB	44.51	8.18	0.422
	non-AB	45.90	11.04	
Low Density Lipoprotein (mg/dl)	AB	104.91	28.89	0.641
	non-AB	107.28	32.24	
Plasma Creatinine (mg/dl)	AB	0.88	0.15	0.78
	non-AB	0.89	0.18	
Hemoglobin (mg/dl)	AB	12.44	1.16	0.454
	non-AB	12.28	1.31	
FBS (mg/dl)	AB	135.52	46.04	0.633
	non-AB	139.65	54.27	
HbA1C (%)	AB	7.52	1.40	0.095
	non-AB	7.94	1.54	
TSH (Iu/l)	AB	2.70	1.45	0.909
	non-AB	2.67	1.84	
Plasma Creatinine (mg/dl)	AB	0.88	0.15	0.78
	non-AB	0.89	0.18	

DM: diabetes mellitus; DBP: diastolic blood pressure; SBP: systolic blood pressure; HbA1c: hemoglobin A1c; TSH: thyroid stimulating hormone; BMI: body mass index.

## Discussion

Researchers have been trying to evaluate the association between ABO blood groups and various disorders for years; including studies to determine the association between ABO group and the development of CHD. However, there are currently no study on diabetic patients addressing the correlation between ABO blood groups and the development of CHD. The present study, to the best of our knowledge, is the first study that focuses on diabetic women and shows a correlation between AB blood group and the development of CHD.

Non-O blood groups have been introduced as a CHD risk factor, and it has been suggested that people with the non-O blood group have more serious coronary artery stenosis than those with O blood group^16^.

In a prospective, five-year study conducted by Medalie and colleagues^17^, among 10,000 males, subjects with blood group A, B, and AB experienced higher rates of myocardial infarction than those with other blood groups. There is no consensus regarding the importance of non-O blood groups (A, B, or AB) in the risk of CHD development. Some researchers have suggested the A blood group to be a CHD risk factor^18,19^, while others have suggested the B and AB blood groups to be risk factors^20,21^. In addition, none of the above studies focused on diabetic patients and more specifically on diabetic women. In study conducted by Meade and colleagues^21^, among 1393 male subjects, the blood groups of subjects were determined, and they were followed for about 16 years. Similar to our study, they reported significantly higher incidences of CHD among the individuals with AB blood group than those with non-AB blood groups. In addition, a combined analysis of two large, prospective cohort studies (the Nurses’ Health Study [NHS] including 62,073 women and the Health Professionals Follow-up Study [HPFS] including 27,428 men), the incidence rate of CHD per 100,000 person/years was higher in people with non-O blood groups^22^. Compared with individuals with O blood groups, individuals with blood groups A, B, or AB had a 5%, 11%, and 23% increased risk of developing CHD in an age-adjusted model, respectively. The adjusted hazard ratios [95% CI] for the incidences of CHD in participants with blood groups A, B, or AB, compared with participants with blood group O, were 1.06 [0.99–1.15], 1.15 [1.04–1.26], and 1.23 [1.11–1.36]; respectively.

The blood group antigens A, B, O have been introduced as a probable independent risk factor for CHD development^18,23^. The association between ABO blood groups and CHD development can be explained through different mechanisms such as higher incidence of thrombotic events, higher systolic and diastolic blood pressure and/or worse blood lipid profile.

In a cohort study, on the 64,686 blood donors aged ≥18 years in Quebec, thrombotic events were higher in subjects with blood group AB compared to blood group O, OR = 1.19 (95% CI: 1.01–1.40)^24^. They showed that the events were higher in women aged ≥40 years with A blood group. In the Framingham heart cohort study^25^, non-O blood groups, especially A blood group, were associated with CHD development. Various factors are considered for the development of thrombus in some blood groups.The higher levels or functional modifications of specific endothelial-derived glycoproteins (GPs), specific platelet GPs, and GPs from either source, and/or GPs from additional cells and tissues, might attribute to the increased risk of thrombosis associated with non-O blood groups^26^. Some investigators have reported that elevated plasma levels of Factor VIII and the Von Willebrand factor (VWF) are associated with the higher prevalence rate of thrombotic events^17^. In addition, several studies have demonstrated that patients with AB blood group have the highest levels of VWF, which is associated with thrombus formation^27^. Some other reports have shown that VWF levels are lower among patients with O blood group versus non-O groups^28^. Furthermore, another study has noted that the plasma half-life of VWF was significantly shorter in patients with the O blood group than the non-O groups^29^.

Kesteloot and colleagues^30^ reported patients with AB blood group to have higher systolic and diastolic blood pressure rates compared with non-AB individuals. Additionally, the role of systolic blood pressure in increasing the risk of CHD has been well documented by various other studies^31,32^.

Number of investigations has noted a positive association between high serum cholesterol levels and CHD in patients with non-O blood groups than those with O blood group^22,33^. Biswas and colleagues evaluated the distribution of ABO blood groups in CHD patients in India, and reported that the risk of cardiovascular disease in AB blood group is higher than those with other groups^34^. They attributed this finding to a lower concentration of high-density lipoprotein cholesterol (HDL-c) in patients with the O blood group and a higher concentration in individuals with the AB blood group, respectively.

In our study, the systolic blood pressure was higher in the AB blood group type, although this difference was not statistically significant (P = 0.065). In addition, lipid profiles were also comparable between patients with AB and non-AB blood groups.

This discrepancy between our results in diabetic women and the aforementioned findings in general population could be explained by the intensive care given to the diabetic patients to control their lipid profile and blood pressure. They often take lipid lowering and/or antihypertensive drugs for some other comorbidity, such as albuminuria, in spite of their normal blood pressures or lipid profiles. One limitation of our study is the lack of data on plasma levels and half-life of VWF, which could provide a more clear association between AB blood group and CHD as mentioned in other studies^28,35^.

To the best of our knowledge, this is the first case-control study in women with T2DM who evaluates the correlation between the risk of CHD development and various blood groups, in which all patients were matched for baseline and demographic variables.

In summary, our current study demonstrates an association between CHD development and AB blood group in women with T2DM. Therefore, we recommend considering AB blood group as another CHD risk factor in daily clinical practice.

More comprehensive studies are required to confirm the role of the AB blood group type in CHD in diabetic patients.

### What is already known on this subject?

While there are several studies on the effect of ABO blood group on coronary heart disease, to the best of our knowledge, this is the first study on T2DM women. Additionally, we went to great lengths to match coronary heart disease risk factors between our cases and controls.

### What does this study add?

We found that the percentage of the AB blood group was significantly higher in T2DM women with concurrent CHD than in those without coronary heart disease.

## References

[1] Almdal T, Scharling H, Jensen JS, Vestergaard H (2004). The independent effect of type 2 diabetes mellitus on ischemic heart disease, stroke, and death: a population-based study of 13,000 men and women with 20 years of follow-up. Arch Intern Med.

[2] Kashi Z (2015). Ischemic Heart Disease and Related Factors in Patients with Diabetes Mellitus Type II. Journal of Mazandaran University of Medical Sciences.

[3] Huxley R, Barzi F, Woodward M (2006). Excess risk of fatal coronary heart disease associated with diabetes in men and women: meta-analysis of 37 prospective cohort studies. Bmj.

[4] Gregg, E. W., Gu, Q., Cheng, Y. J., Narayan, K. M. & Cowie, C. C. Mortality trends in men and women with diabetes, 1971 to 2000. *Ann Intern Med* **147**, 149–155 (2007). 10.7326/0003-4819-147-3-200708070-00167 17576993

[5] Zhang X (2017). Gender Disparities in Lipid Goal Attainment among Type 2 Diabetes Outpatients with Coronary Heart Disease: Results from the CCMR-3B Study. Scientific reports.

[6] Niskanen L, Turpeinen A, Penttila I, Uusitupa MI (1998). Hyperglycemia and compositional lipoprotein abnormalities as predictors of cardiovascular mortality in type 2 diabetes: a 15-year follow-up from the time of diagnosis. Diabetes Care.

[7] Yang ZJ (2012). Prevalence of cardiovascular disease risk factor in the Chinese population: the 2007-2008 China National Diabetes and Metabolic Disorders Study. Eur Heart J.

[8] Leon BM, Maddox TM (2015). Diabetes and cardiovascular disease: Epidemiology, biological mechanisms, treatment recommendations and future research. World J Diabetes.

[9] Peters SA, Huxley RR, Woodward M (2014). Diabetes as risk factor for incident coronary heart disease in women compared with men: a systematic review and meta-analysis of 64 cohorts including 858,507 individuals and 28,203 coronary events. Diabetologia.

[10] Edgren G (2010). Risk of gastric cancer and peptic ulcers in relation to ABO blood type: a cohort study. Am J Epidemiol.

[11] Wang Z (2012). ABO blood group system and gastric cancer: a case-control study and meta-analysis. Int J Mol Sci.

[12] Harris JB (2005). Blood group, immunity, and risk of infection with Vibrio cholerae in an area of endemicity. Infect Immun.

[13] langari H, Kashi Z, asadian L, Abediankenari S, Bahar A (2016). ABO and Rh Blood Groups in Type 2 Diabetes Mellitus, North of Iran. Journal of Mazandaran University of Medical Sciences.

[14] Fihn SD (2012). 2012 ACCF/AHA/ACP/AATS/PCNA/SCAI/STS guideline for the diagnosis and management of patients with stable ischemic heart disease: executive summary: a report of the American College of Cardiology Foundation/American Heart Association task force on practice guidelines, and the American College of Physicians, American Association for Thoracic Surgery, Preventive Cardiovascular Nurses Association, Society for Cardiovascular Angiography and Interventions, and Society of Thoracic Surgeons. Circulation.

[15] Fihn SD (2014). 2014 ACC/AHA/AATS/PCNA/SCAI/STS focused update of the guideline for the diagnosis and management of patients with stable ischemic heart disease: a report of the American College of Cardiology/American Heart Association Task Force on Practice Guidelines, and the American Association for Thoracic Surgery, Preventive Cardiovascular Nurses Association, Society for Cardiovascular Angiography and Interventions, and Society of Thoracic Surgeons. Journal of the American College of Cardiology.

[16] Huang X (2017). Relation of ABO Blood Groups to the Plaque Characteristic of Coronary Atherosclerosis. Biomed Res Int.

[17] Franchini M, Mannucci PM (2014). ABO blood group and thrombotic vascular disease. Thromb Haemost.

[18] Zhou S, Welsby I, Is ABO (2014). blood group truly a risk factor for thrombosis and adverse outcomes?. World J Cardiol.

[19] Whincup PH, Cook DG, Phillips AN, Shaper AG (1990). ABO blood group and ischaemic heart disease in British men. Bmj.

[20] Nydegger UE, Wuillemin WA, Julmy F, Meyer BJ, Carrel TP (2003). Association of ABO histo-blood group B allele with myocardial infarction. Eur J Immunogenet.

[21] Meade TW (1994). Factor VIII, ABO blood group and the incidence of ischaemic heart disease. Br J Haematol.

[22] He M (2012). ABO blood group and risk of coronary heart disease in two prospective cohort studies. Arterioscler Thromb Vasc Biol.

[23] Chen Z, Yang SH, Xu H, Li JJ (2016). ABO blood group system and the coronary artery disease: an updated systematic review and meta-analysis. Scientific reports.

[24] Blais C, Germain M, Delage G, Gregoire Y (2016). The association between blood group and the risk of vascular disease in Quebec blood donors. Blood Transfus.

[25] Garrison RJ (1976). ABO blood group and cardiovacular disease: the Framingham study. Atherosclerosis.

[26] Zhang H, Mooney CJ, Reilly MP (2012). ABO Blood Groups and Cardiovascular Diseases. Int J Vasc Med.

[27] Moeller A, Weippert-Kretschmer M, Prinz H, Kretschmer V (2001). Influence of ABO blood groups on primary hemostasis. Transfusion.

[28] Jenkins PV, O’Donnell JS (2006). ABO blood group determines plasma von Willebrand factor levels: a biologic function after all?. Transfusion.

[29] Vlot AJ (2000). The half-life of infused factor VIII is shorter in hemophiliac patients with blood group O than in those with blood group A. Thromb Haemost.

[30] Kesteloot H, Van Houte O (1974). An epidemiologic survey of arterial blood pressure in a large male population group. Am J Epidemiol.

[31] Sarnak MJ (2003). Kidney disease as a risk factor for development of cardiovascular disease: a statement from the American Heart Association Councils on Kidney in Cardiovascular Disease, High Blood Pressure Research, Clinical Cardiology, and Epidemiology and Prevention. Hypertension.

[32] Lieb W (2013). Genetic predisposition to higher blood pressure increases coronary artery disease risk. Hypertension.

[33] Chen Y (2014). Analysis of circulating cholesterol levels as a mediator of an association between ABO blood group and coronary heart disease. Circ Cardiovasc Genet.

[34] Biswas S, Ghoshal PK, Halder B, Mandal N (2013). Distribution of ABO blood group and major cardiovascular risk factors with coronary heart disease. Biomed Res Int.

[35] Sari I (2008). ABO blood group distribution and major cardiovascular risk factors in patients with acute myocardial infarction. Blood Coagul Fibrinolysis.

